# Supplementary material for: Neuronal parts list and wiring diagram for a visual system
Source: Nature. 2024 Oct 2;634(8032):166–80. doi: 10.1038/s41586-024-07981-1 (PMC11446827; doi:10.1038/s41586-024-07981-1)
Supplement: Supplementary file 5 — Cards for all cell types. Each card has 6 panels (clockwise): logical connectivity predicate diagram, numeric stats, illustration of a typical cell, followed by 3D rendering of all cells from three orthogonal viewpoints. Also available at Codex. [file 41586_2024_7981_MOESM5_ESM.zip › pdf/Dm16.pdf]

*Dm*16

predicate precision: 96%, recall: 98%

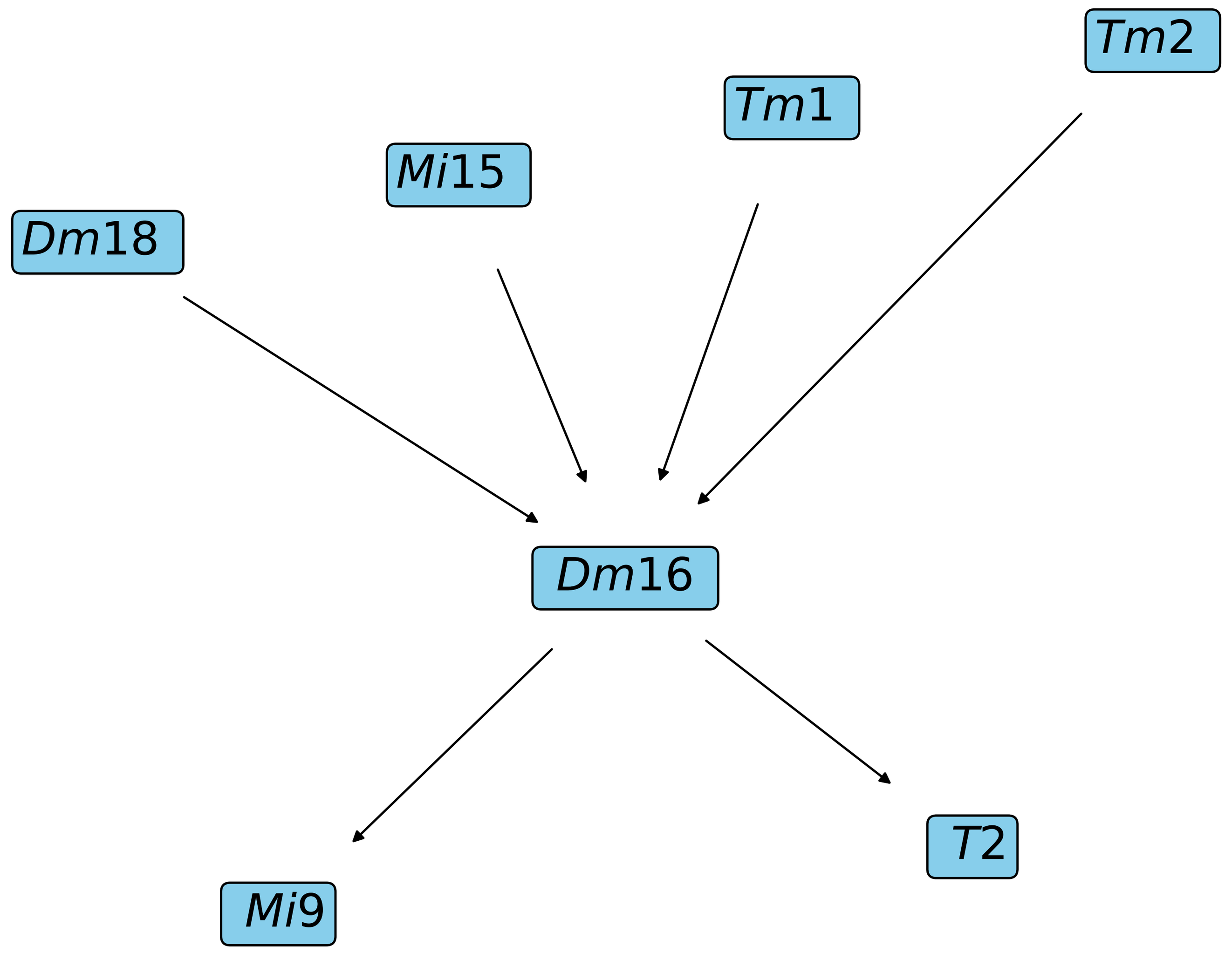

avg. stats

cells	44
NT prediction	GABA (50%)
top in/out regions	ME (99%) / ME (99%)
in/out degree	59 / 61
in/out synapses	192 / 192
cable len	935μ
surface	$1,281\mu^2$
volume	$73\mu^3$

morphology

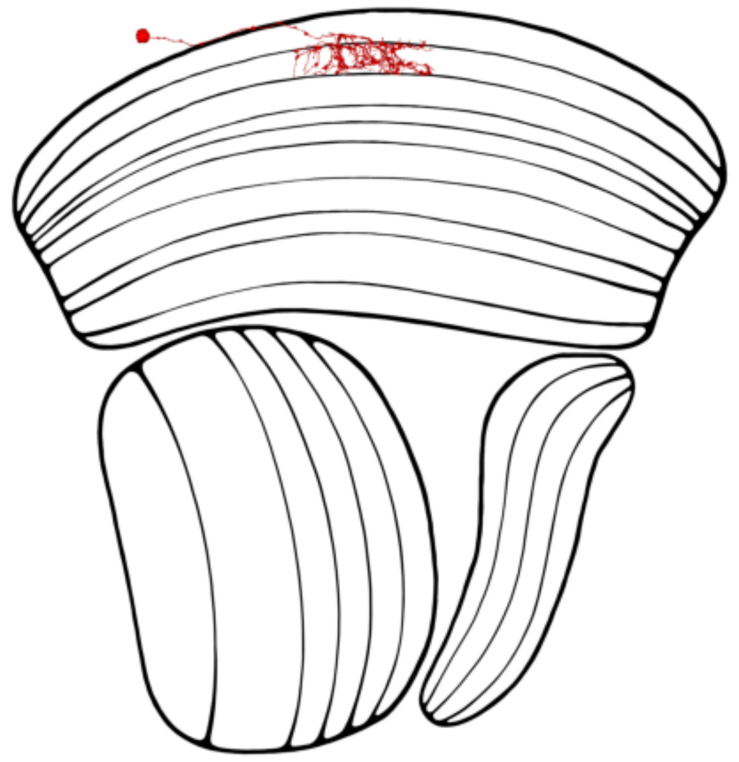

anterior view

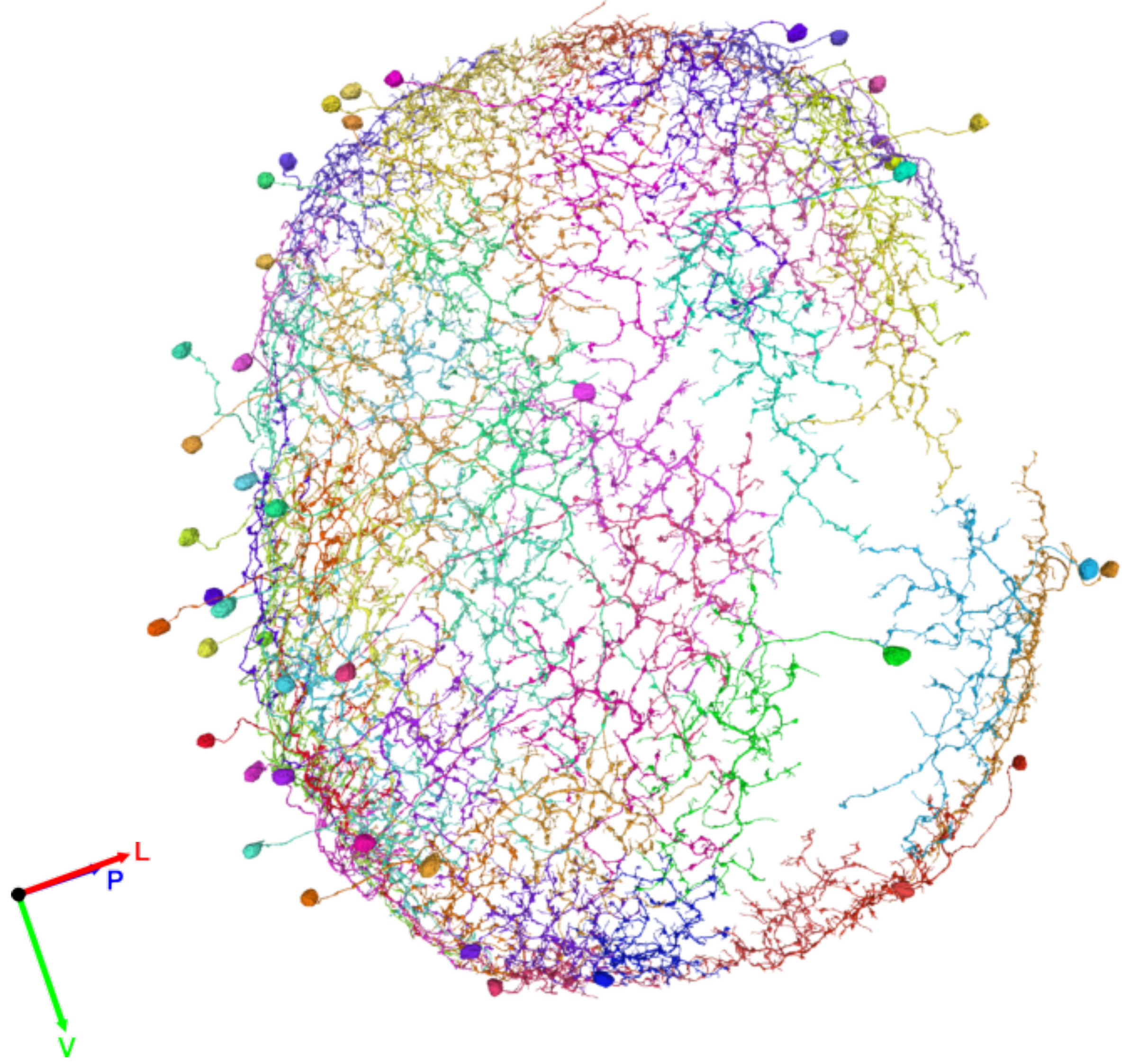

ventral view

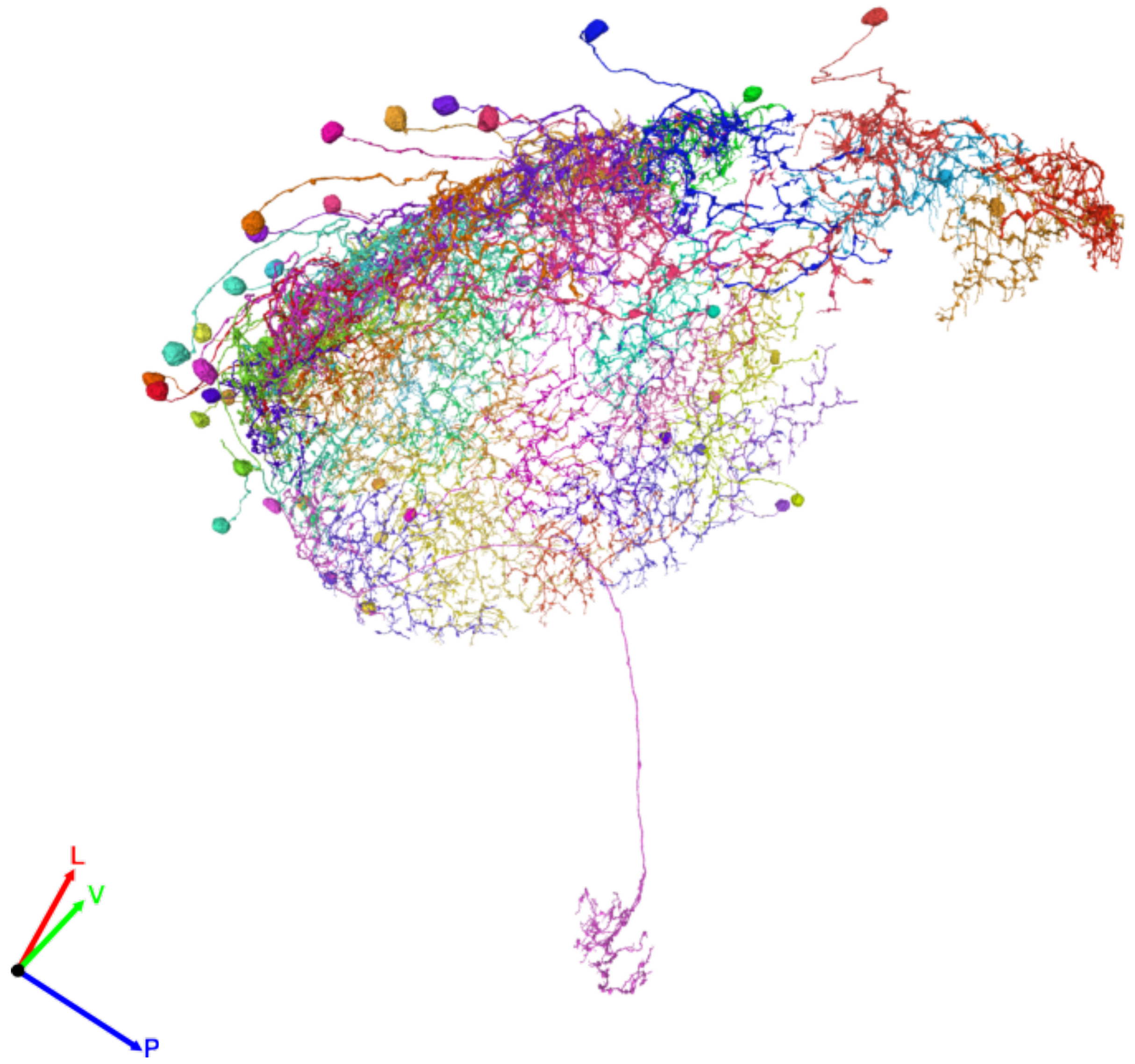

side view

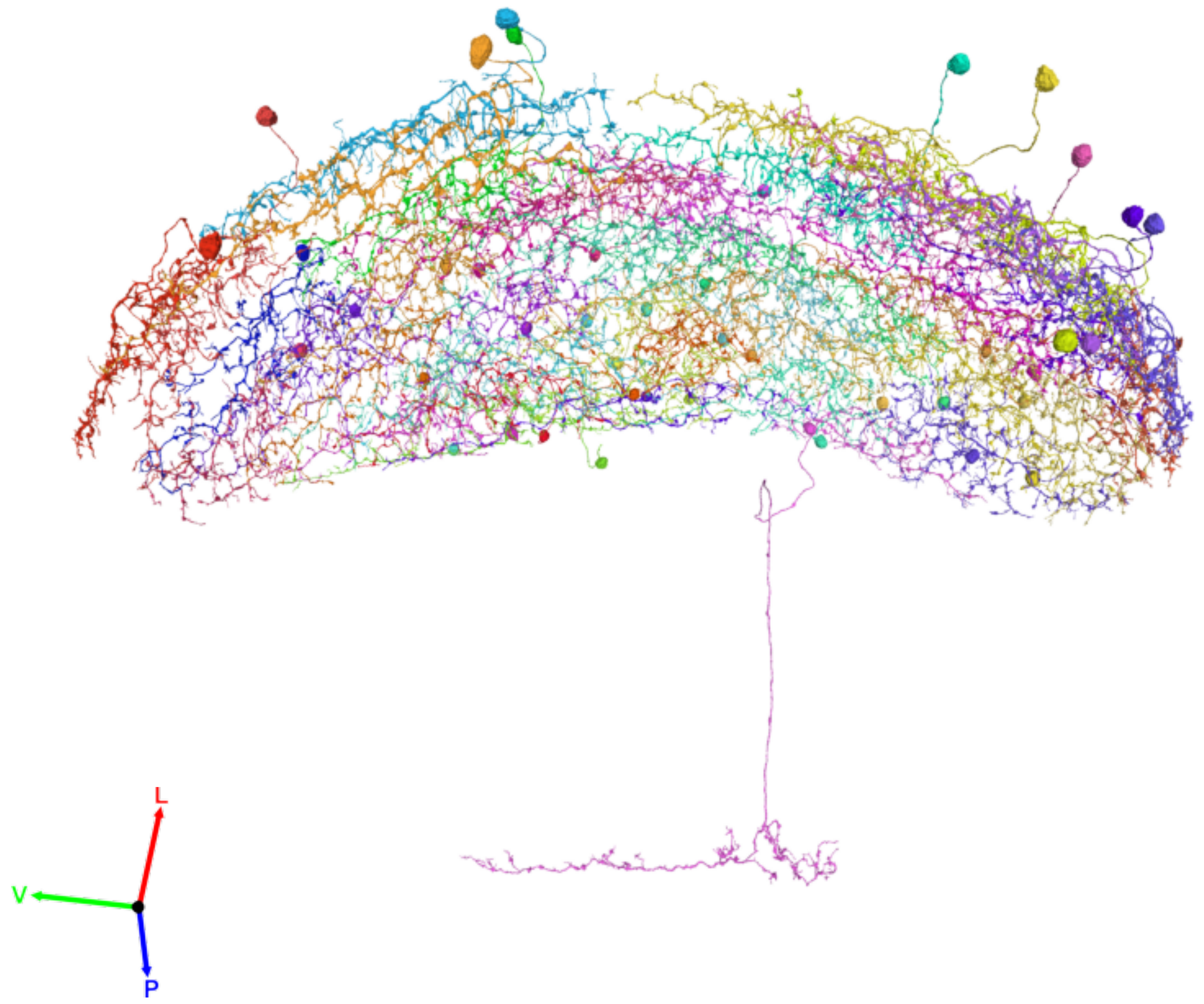

Distal Medulla
